# Sensitive and In Situ Hemoglobin Detection Based on a Graphene Oxide Functionalized Microfiber

**DOI:** 10.3390/nano10122461

**Published:** 2020-12-09

**Authors:** Fang Fang, Yanpeng Li, Liuyang Yang, Liangye Li, Zhijun Yan, Qizhen Sun

**Affiliations:** School of Optical and Electronic Information & National Engineer Laboratory for Next Generation Internal Access System & Wuhan National Laboratory for Optoelectronics, Huazhong University of Science and Technology, Wuhan 430074, China; ffsherry@hust.edu.cn (F.F.); yanpli@hust.edu.cn (Y.L.); lyyang@hust.edu.cn (L.Y.); liangyeli@hust.edu.cn (L.L.); yanzhijun@mail.hust.edu.cn (Z.Y.)

**Keywords:** graphene oxide, microfiber, hemoglobin

## Abstract

The determination of hemoglobin (Hb) level is indispensable in the pathological study of many blood diseases. Graphene oxide (GO), with its excellent optical properties and great biocompatibility, has attracted significant attention and been widely utilized in biochemical detection. Here, we report an ultrasensitive Hb sensor based on a graphene oxide (GO)-coated microfiber. The GO was utilized as a linking layer deposited on the microfiber surface, which can provide an enhanced local evanescent light field and abundant bonding sites for Hb molecules. The optical microfiber with a compact structure and a strong evanescent light field served as the platform for biosensing. The surface morphology characterized by optical microscope, scanning electron microscope, and Raman spectroscopy offers detailed evidence for the success of GO deposition. The dynamic bonding between GO and target Hb molecules was monitored in real-time through an optical spectrum analyzer. An ultrahigh sensitivity of 6.02 nm/(mg/mL) with a detection limit of 0.17 μg/mL was achieved by tracking the resonant wavelength shift of spectra. It is important to highlight that the detection limit of GO-coated microfiber is 1–2 orders of magnitude lower than other reported fiber optic Hb sensors. Benefiting from high sensitivity, low cost, small size, and fast response, the proposed sensing microfiber coated with GO could be a competitive alternative in the diagnosis of blood diseases and a subject of further research in the medical field.

## 1. Introduction

The determination of hemoglobin (Hb) level plays an important role in the pathological study of many diseases, such as hereditary anemia [1,2], polycythemia vera [3], hematuria [4], and acute gastrointestinal bleeding [5]. Specifically, hematuria is a clinical symptom of chronic nephritis, kidney stone, and renal carcinoma. Moreover, acute gastrointestinal bleeding can bring about serious consequences, including hemorrhagic shock, hematemesis, and other severe physical conditions [6]. Early recognition and fast detection of Hb in medical treatments can effectively decrease pathological blood loss and further reduce the risk for patients. Considering the significance of accurate quantitative analysis of Hb levels, numerous methods have been developed to determine the concentration of Hb, mainly including electrochemistry [7], spectrophotometry [8,9], the chemiluminescence method [10], the colorimetric method [11], etc. However, most of these methods are relatively complicated, time-consuming, and costly, limiting their practical applications. Therefore, the development of simple, efficient, cost-effective, and user-friendly methods for Hb measurement is still in high demand.

Fiber optic sensors have been widely used in the fields of biochemical sensing due to their advantages of high sensitivity, low cost, compact size, and great convenience. In the development of fiber optic biochemical sensors, enhancing the interaction of light with target object is crucial to achieving high sensitivity. For this purpose, researchers have developed many special structures to disturb light propagation, such as U-shaped fibers [12], long period grating (LPG) [13], and titled fiber grating (TFG) [14,15]. For instance, Deep et al. proposed a LPG-based glucose sensor with the sensitivity of 0.806 nm/(mg/mL) [16], and Voisin et al. utilized a gold-coated TFG for transferrin detection with the limit of 1 μg/mL [17]. However, owing that the optical field is mainly confined to the fiber core, most of the fiber biochemical sensors suffer from relatively low sensitivity, making it difficult for them to realize the micro-determination of Hb level. Optical microfiber, with its intrinsically strong evanescent field, provides an excellent platform for light-matter interaction, which could realize fast and highly sensitive detection of biochemicals at low volume level [18,19]. Currently, microfiber has been utilized in the detection of proteins, enzymes, and bacteria [20,21,22]. At the same time, to achieve the highly sensitive detection of biomolecules, microfiber still needs the help of special materials that can distinguish and capture the target molecules from the external environment.

In recent years, the advancement of 2D-nanomaterials is attracting increasing attention and has been the subject of research in multidisciplinary fields, including biology, chemistry, and physics [23]. Biosensing benefits from nanotechnology due to the unique electronic, optical, and catalytic properties of nanomaterials, which can facilitate the combination and recognition of biomolecules [24]. Among the novel nanomaterials, graphene oxide (GO), an important derivative of graphene, is considered a promising material for biosensing with a large specific area and great biocompatibility [25,26]. Compared with other porous material structures, GO has sp^2^- and sp^3^-hybridized carbon atoms as well as enriched oxygen-containing groups, such as carboxyl, hydroxyl, and carbonyl [27]. The functional groups on GO could not only supply a stable reactive environment for aqueous and polar solvents, but also serve as sites for functionalization. Moreover, biomolecules can be immobilized on the surface of GO through strong noncovalent π-stacking interaction, which is meaningful to achieve sensitive and reliable detection [28,29]. Benefiting from these unique properties, GO is regarded as an ideal material for hemoglobin detection.

**Figure 1:**
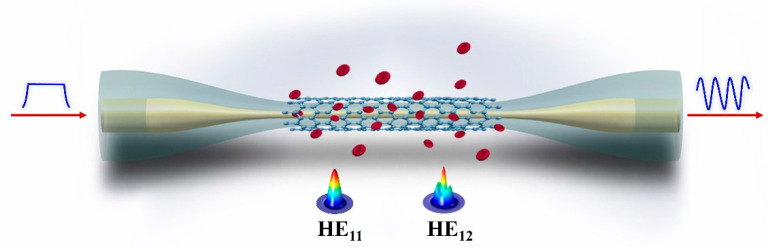
Schematic diagram of the proposed microfiber-based hemoglobin biosensor.

In this study, we innovatively combine the merits of GO and microfiber to achieve the ultrasensitive detection of Hb. The detection mechanism is schematically illustrated in Figure 1. A microfiber mode interferometer is utilized as the sensing structure, which is fabricated through non-adiabatically tapering a single mode fiber (SMF) into micrometer size [30]. In order to efficiently recognize the Hb molecules from the external environment, the GO is deposited on microfiber as the linking layer through chemical bonding and physical absorption. The GO-coated microfiber presents a strong absorbability for Hb molecules. By tracking the spectral variation of microfiber during the dynamic bonding process between the GO and Hb molecules, Hb detection with a high sensitivity of 6.02 nm/(mg/mL) is realized. The proposed method for Hb detection has the advantages of high sensitivity, low cost, and convenient operation, which has great potential for applications in clinical research and disease diagnosis.

## 2. Materials and Methods

### 2.1. Reagents and Materials

The aqueous dispersion of GO, lyophilized powder of human Hb and 3-aminopropyl-triethoxysilane (APTES) were purchased from Sigma-Aldrich (Shanghai, China). Sodium hydroxide (NaOH) was supplied from Sinopharm Chemical Reagent Co., Ltd (Shanghai, China). Polydimethylsiloxane (PDMS) and curing agent were purchased from Wenhao Co., Ltd (Suzhou, China). Deionized water was derived from a milli-Q water purifying system (Wuhan, China). All Hb solutions were prepared with deionized (DI) water.

### 2.2. Fabrication and Refractive Index (RI) Sensitivity of Microfiber

**Figure 2:**
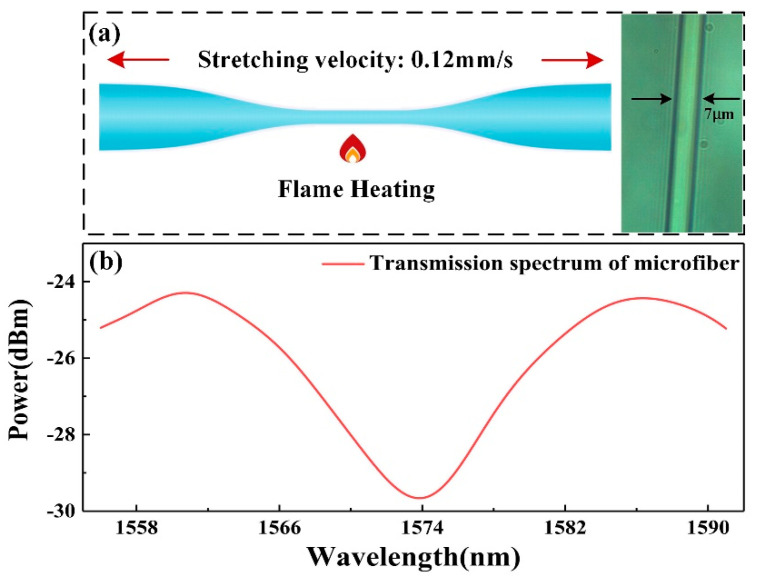
(**a**) Fabrication method and micrograph of microfiber and (**b**) the reflective spectrum of the microfiber in water.

The sensing microfiber was fabricated by a flame heating-drawing system, which included a heating source and an electric displacement platform. Different fiber length and diameter could be obtained through controlling the mass flow of oxyhydrogen flame and stretching velocity of the displacement platform. A multimode microfiber (MMMF) was non-adiabatically drawn from a standard single mode fiber with the velocity of 0.12 mm/s. The multimode microfiber was utilized as a transducer to detect the surrounding RI change near the fiber surface induced by Hb biomolecular binding interaction. To achieve a high RI sensitivity, the diameter of multimode microfiber should be close to the dispersion turning point, according to our previous work [30]. Considering that the microfiber with an excessively small diameter is fragile, the waist diameter of microfiber in this work was chosen to be about 7 μm, as shown in Figure 2a. The RI sensitivity was independent of the length of the microfiber [30], and thus the sensing length of the microfiber utilized in this work was ~4.5 mm, which was enough for functionalization and Hb sensing. The transmission loss of microfiber could be reduced by less than 0.1 dB/mm by optimizing the fabrication method, which could be neglected in the experiment. When the light was guided into the microfiber, the fundamental mode and higher order modes (mainly HE_11_ and HE_12_ modes) became excited and generated the mode interference. The spectrum of obtained microfiber was monitored by the optical spectrum analyzer (OSA). From the result presented in Figure 2b, it can be seen that a clear interference spectrum was generated with the extinction ratio higher than 5 dB.

**Figure 3:**
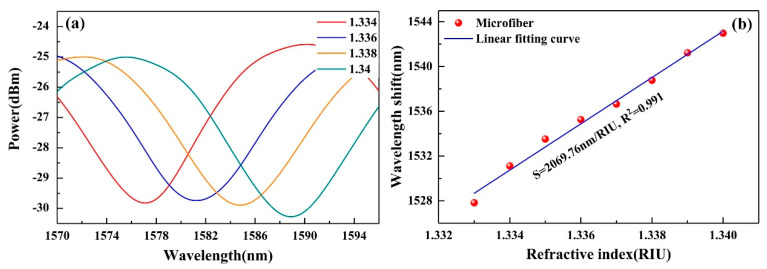
RI sensitivity of the microfiber: (**a**) transmission spectra in different RI solutions and (**b**) relationship between the wavelength shift and the RI.

Through immersing the microfiber into a set of prepared aqueous glycerol solutions with the RI ranging from 1.333 to 1.34, the RI sensitivity was investigated. The monitored transmission spectra are shown in Figure 3a. For clearly observing the resonant wavelength shift of different concentrations, only four representative spectra are presented. It is obvious that the resonant dip shifts to longer wavelength when the RI increases. The relationship between wavelength shifts and RI is plotted in Figure 3b, which shows that the RI sensitivity of the microfiber is ~2069.76 nm/RIU with a good linearity of 0.991.

### 2.3. Surface Functionalization and Characterization of Microfiber

**Figure 4:**
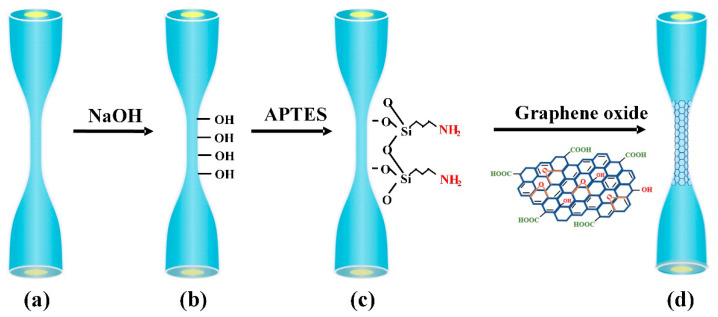
Schematic illustration of functionalization process: (**a**) HNO_3_-cleaned, (**b**) Hydroxyl-groups activated, (**c**) APTES-modified, and (**d**) GO-deposited microfiber.

To achieve highly sensitive detection of Hb, the surface modification with GO is indispensable. GO was deposited on microfiber through chemical bonding and physical absorption. Figure 4a–d schematically illustrate the functionalization process. Firstly, it was immersed in 5% HNO_3_ for 2 h at room temperature and then washed by deionized water and ethanol several times to remove the surface residues. After cleaning, the microfiber was immersed in 1.0 M NaOH solution for 1 h to activate the hydroxyl (-OH) groups on the surface (see Figure 4b), then washed by deionized water thoroughly and dried naturally. Next, the microfiber was left in 3-aminopropyl-triethoxysilane (APTES) (5% *v*/*v* in ethanoic solution) for 30 min. During the process, the amidogen (-NH_2_) was formed on the fiber surface through Si-O-Si bonding (see Figure 4c). After that, the microfiber was cleaned with ethanol to remove the unbound molecules. After APTES silylanization, the microfiber was dipped in 2 mg/mL GO aqueous solution for 3 h. It is noted that GO solvents was centrifuged with 4000 r/min in advance to obtain homogeneous GO suspension for better bonding. The epoxy groups of GO will react with the bonded amino groups on fiber surface to form GO layer gradually (see Figure 4d). The GO coating layer was stable and hard to remove in a general experimental environment. After GO deposition, the microfiber was washed several times with DI water to remove the surface residues. When the deposition process finishes, a brownish coating can be achieved on the surface of microfiber.

**Figure 5:**
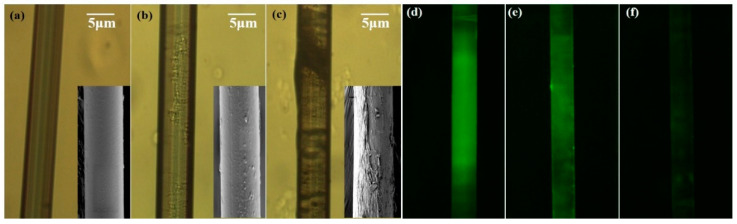
Microscope images and SEM images of (**a**) bare microfiber, (**b**) APTES-modified microfiber, and (**c**) GO-deposited microfiber; fluorescence images of (**d**) GO-coated microfiber, microfiber after reaction with (**e**) 0.2 mg/mL and (**f**) 1 mg/mL Hb solutions.

After functionalization, the surface morphology of the microfiber is characterized by optical microscope (Sunny Optical Technology Co., Ltd, Yuyao, China). Figure 5a–c present the surface micrographs of the bare microfiber, APTES-modified microfiber, and GO-coated microfiber, respectively. Compared with Figure 5a, it can be found that the fiber surface appears relatively rough after APTES silylanization. To observe the fiber surface more clearly, micrographs with higher resolution were also taken by a scanning electron microscope (GeminiSEM, ZEISS international Inc., Oberkochen, Germany), which are illustrated in the inset of Figure 5a–c. The speckles on the APTES-coated microfiber result from the uneven coating of APTES. Additionally, the black coating and wrinkled porous layers exhibited in Figure 5c and its inset indicate that the GO has been bonded onto the fiber surface successfully. In order to verify that the target Hb molecules could be recognized and interact with the coated GO, a confirmatory experiment was conducted. Figure 5d–f show the fluorescence images of microfibers under different Hb concentrations. The images were taken by an inverted fluorescent microscope with the excitation wavelength of 460 nm (Olympus IX70, Olympus Corporation Inc., Tokyo, Japan). It can be seen in Figure 5d that strong fluorescence appears when the GO is coated on the microfiber surface. The fluorescence originates from the electronic transitions between the non-oxidized carbon regions and the boundary of oxidized carbon atom regions [31]. After the interaction between the GO on the microfiber and the Hb solution with the concentration of 0.2 mg/mL, the fluorescence signal becomes weaker (see Figure 5e). While the microfiber is immersed into the Hb solution with the higher concentration of 1 mg/mL, the fluorescence signal almost disappears (see Figure 5f). Therefore, as the Hb concentration increases, the fluorescence signal is weakened by the reduction of related fluorescent groups. The above phenomenon indicates that the GO-coated microfiber has the capacity to recognize the Hb samples with different concentrations.

**Figure 6:**
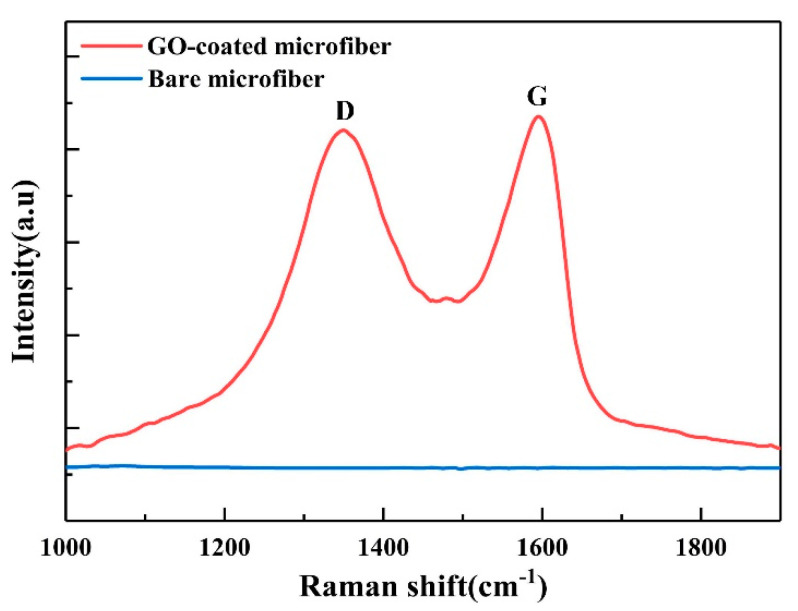
Raman spectra of bare and GO-coated microfiber.

Raman spectroscopy is a widely used tool for the characterization of carbon-based materials. Both GO-coated and bare microfiber are characterized by a laser confocal Raman spectrometer (LabRAM HR800, HORIBA Inc., Paris, France) with 532 nm laser excitation. The Raman spectra are depicted in Figure 6. Compared with bare microfiber, two prominent peaks presented with red curve could be observed around 1350 cm^−1^ and 1598 cm^−1^, corresponding to the D and G Raman modes of graphitic carbon sheet, respectively. The D band is induced by the ring vibration symmetrical breathing mode and associated with the defects caused by the attachment of hydroxyl and epoxide groups. The G band reflects the first-order scattering of the E_2g_ plane of sp^2^ carbon atoms [32]. The above characterizations reveal that the GO is effectively deposited on the microfiber surface.

## 3. Experimental Setup

### 3.1. Encapsulation of Microfiber

**Figure 7:**
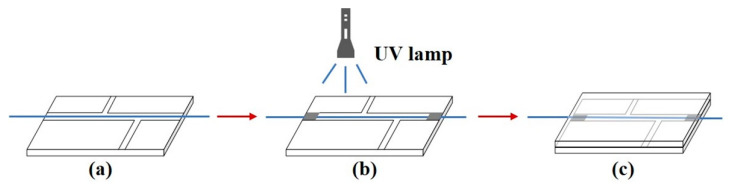
Schematic illustration of encapsulation process: (**a**) embedding the microfiber in the microchannel, (**b**) immobilizing the microfiber by UV photoresist and sealing the horizontal microchannel, and (**c**) adhering another polydimethylsiloxane (PDMS) block onto the first one.

Note that most of the previously reported microfiber-based biosensors are suspended in air or mounted in an open experimental system. The stability of these sensors is affected by surface contamination and environment factors, resulting in inestimable measurement error. In order to create a more stable sensing environment and control the reagents’ dosage precisely, the GO-coated microfiber was encapsulated in a microfluidic chip to isolate it from external environment and make it more suitable for practical Hb sensing. As a well-accepted material with excellent biocompatibility, PDMS was chosen as the chip material. The packaging method is schematically depicted in Figure 7. Firstly, the microfiber was embedded into the horizontal microchannel of one block of customized PDMS as shown in Figure 7a. After that, the microfiber was fixed by ultraviolet (UV) photoresist and the horizontal microchannel was sealed simultaneously. At this moment, only liquid channels were open (see Figure 7b). Finally, another block of PDMS was covered and adhered onto the first one firmly to avoid the leakage of reagents (see Figure 7c). During the sensing experiment, the Hb solutions were injected and extracted through the inlet and outlet microchannels of the microfluidic chip.

### 3.2. The Measurement System for Hb Detection

**Figure 8:**
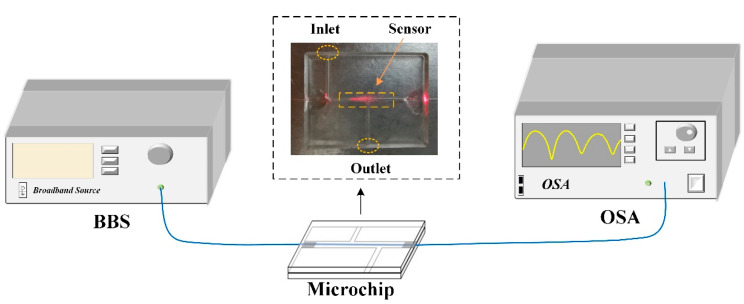
The experimental setup for Hb detection.

The schematic diagram of the measurement system for Hb detection is illustrated in Figure 8. A broadband source (BBS) was used to launch the light to sensor, and the transmission spectra were monitored by the OSA in real-time. The Hb solutions with different concentrations were prepared by dissolving the stock Hb powder in deionized water. In this study, the concentrations of Hb solutions ranged from 0 to 1 mg/mL with a step of 0.1 mg/mL. During the biosensing process, Hb solutions were injected into the sensing chip and resonant wavelength shifts were tracked continuously. After each measurement, the reagent was extracted out of the chip and microfiber was cleaned with deionized water for next test. In addition, the experimental temperature was kept at ~20 °C to exclude the effect of the temperature fluctuation.

## 4. Results and Discussion

**Figure 9:**
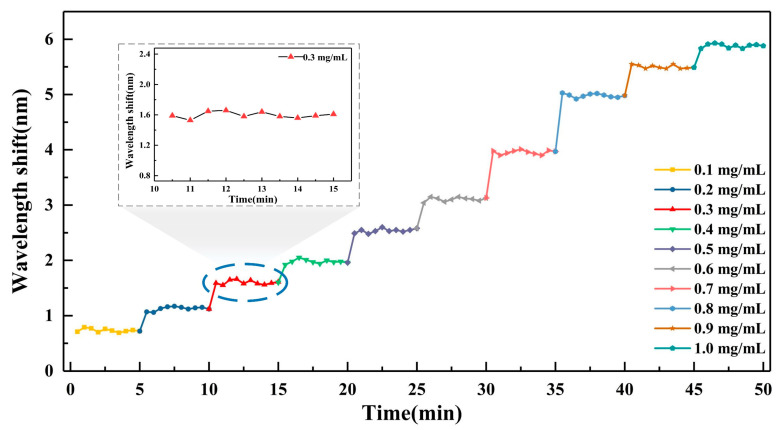
The real-time wavelength shift responses of microfiber in Hb detection. Inset: transmission spectral responses at 0.3 mg/mL.

In order to test the stability of the GO-coated microfiber in the measurement, the transmission spectral responses were monitored in real-time for Hb solutions with different concentrations. Figure 9 shows the spectral variation in different concentration of Hb solutions. It can be easily observed that with the increase of Hb concentration from 0 to 1 mg/mL, the resonant dip shifts to larger wavelength regularly. At each measurable concentration, the wavelength shifts change dramatically in the beginning and then tend to a steady state. It can be concluded that the combination between GO and Hb molecules is rapid and then reaches a dynamic equilibrium. The settling time of equilibrium is less than 1 min. The dynamic-equilibrium process in different Hb solutions is monitored and exhibited with different colors and labels. When the combination reaches dynamic equilibrium, the wavelength shift has slight fluctuation due to the influence of the external environment. The maximum fluctuation during the measurement occurs at 0.3 mg/mL, which is only about 0.13 nm (see the inset of Figure 9).

**Figure 10:**
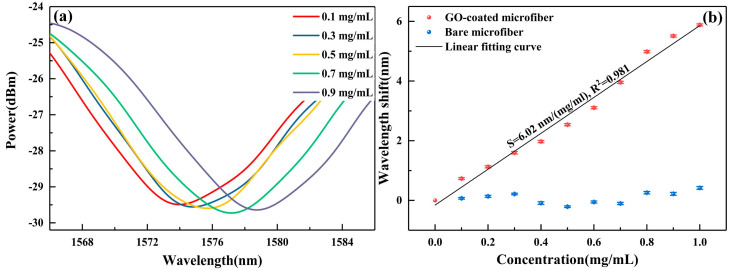
(**a**) Spectra of Hb solutions with different concentration, (**b**) the corresponding wavelength shift with the Hb concentration.

The spectra variations corresponding to different Hb concentrations were monitored by tracking the wavelength shift of resonant dip at ~1574 nm with high extinction ratio over 5 dB. The transmission spectra were collected and depicted in Figure 10a when the shifts of resonant dip were stable at different concentrations. For clear graphical illustration, only five representative concentrations are plotted. It is obvious that when the concentration increases, the resonant dip shifts to a longer wavelength. The wavelength shifts of resonant dip could be attributed to the local refractive index change, which is caused by the π-π interaction between GO and Hb molecules at the fiber interface. The correlation between wavelength shifts and Hb concentrations are illustrated in Figure 10b. In the concentration range of 0–1 mg/mL, the wavelength shifts of GO-coated microfiber increase sequentially and exhibit a linear response to Hb concentrations. From the linear fitting result, an ultra-high sensitivity of 6.02 nm/(mg/mL) with the R-square value of 0.981 is obtained. To fully reveal the sensitivity enhancement of GO for Hb detection, a contrast experiment based on bare microfiber was also conducted. The experimental results are exhibited in Figure 10b as well. For bare microfiber, the maximum variation of resonant wavelength shift is only about 0.47 nm, which is much less than that of GO-coated microfiber. From comparing results between the GO-coated and bare microfiber, it could be seen that the Hb detection sensitivity is significantly enhanced by the deposition of GO.

Table 1 presents the Hb sensing performance in comparison with the previous study of Hb sensors. Compared to the intensity demodulation utilized in the LPG-based Hb sensor [29], which is vulnerable to power fluctuation, the wavelength demodulation used in our study is more reliable and accurate for Hb measurement. Remarkably, the GO-coated microfiber achieves a high sensitivity of 6.02 nm/(mg/mL), which could be ascribed to the strong evanescent field of microfiber and great biocompatibility of GO coating. Hence, the detection limit of the proposed sensor is as low as 0.17 μg/mL, corresponding to the wavelength resolution of 1 pm, which is 1–2 orders of magnitude lower than other reported fiber optic Hb sensors. Although the lowest detectable concentration of the ferrocenoyl cysteine conjugates modified electrode [33] is smaller than observed in our study, the microfiber has the significant advantages of electromagnetic interference immunity, compact size, and long life-time, which could be widely utilized in various application environments. Furthermore, the length and diameter of microfiber could be precisely controlled through regulating the mass flow of oxyhydrogen flame and stretching the velocity of the displacement platform. The GO deposition could be precisely controlled by vapor deposition and self-assembly method. Therefore, the Hb sensors with consistent performance could be produced for medical disposable use.

The excellent sensing performances of the proposed sensor could be ascribed to the following aspects: (i) The GO utilized as absorption layer of Hb molecules increases the bonding sites and provides great biocompatibility for Hb sensing. After the GO deposition, the RI of cladding material is the equivalent value of silicon and GO in proportion. With the increase of GO thickness, the equivalent RI gradually tends to be 1.7 of GO. Therefore, the local evanescent field will be enhanced with the increase of GO thickness, resulting in the improvement of sensitivity. Subsequently, the sensitivity will reach a maximum value with an optimal GO thickness. (ii) The microfiber with micro-scale diameter provides an extremely strong evanescent field for direct light-matter reaction, making it highly sensitive to surrounding changes. Therefore, the micro-determination of biomolecules could be realized by our proposed sensor.

Notably, the sensor performance can be further improved in several aspects. Firstly, the FWHM of the resonant dip can be narrowed to achieve a higher Q-factor, through increasing the sensing length and decreasing the fiber diameter [30]. However, there is a trade-off between the FWHM of resonant dip and the measurement range, which should be optimized according to the detection requirement. Secondly, the GO coating thickness is relatively uneven due to the deposition method utilized in this study. To precisely control the GO coating thickness, vapor deposition and self-assembly method can be explored in future studies. Finally, Hb sensitivity can be optimized through qualitatively analyzing the thickness of GO and choosing the appropriate value.

## 5. Conclusions

We proposed and demonstrated an ultrasensitive Hb sensor based on a GO-coated microfiber. The sensing structure of microfiber was fabricated by the heating-drawing method, which provides an extremely strong evanescent field for light-matter interaction. The GO was successfully functionalized on the surface of the microfiber through chemical bonding and physical absorption, which exhibit superior performances in enhancing the local evanescent field and providing abundant bonding sites for sensitive Hb detection. The surface characterization of GO-coated microfiber was examined by optical microscope, SEM, and Raman spectroscope, which verified the effective and relatively uniform coating of GO. By tracking the resonant wavelength shift with the Hb concentration, an ultrahigh sensitivity of 6.02 nm/(mg/mL) in the range of 0–1 mg/mL was achieved. The detection limit of the proposed sensor is 0.17 μg/mL, which is much lower than other reported fiber optic Hb sensors. The proposed GO-coated microfiber has the merits of ease of fabrication, high sensitivity, fast response, and is suitable for medical disposable use, making it a promising platform for the diagnosis of blood diseases and a subject of further research in the medical field.

## Figures and Tables

**Table 1:** Comparison of sensing performance between different Hb sensors.

Sensing Structure	Diameter × Length	Sensitivity	Detection Limit (μg/mL)	Type of Sensor	Ref.
Long period grating	125 μm × 15 mm	1.9 dB/(mg/mL)	50	Fiber optic sensor	[29]
chalcogenide fiber	–	6.71 × 10^−4^/(g/dL)	1.8	Fiber optic sensor	[34]
Side-polished fiber	– ×17 mm	0.0025 a.u./(mg/mL)	–	Fiber optic sensor	[35]
Dual-core photonic crystal fiber	– ×3.7 cm	~0.08 nm/(mg/mL)	–	Fiber optic sensor	[36]
Nanoparticles modified electrode	4 mm × 4 mm	0.17 mA/(mg/mL)	1	Electrochemical sensor	[37]
Ferrocenoyl cysteine conjugates modified electrode	–	0.4 mA/(mg/mL)	0.03	Electrochemical sensor	[33]
GO-coated microfiber	7 μm × 6.5 mm	6.02 nm/(mg/mL)	0.17	Fiber optic sensor	Our study
